# Supplementary material for: Risk factors affecting polygenic score performance across diverse cohorts
Source: medRxiv. 2024 Apr 10:2023.05.10.23289777. Preprint. [Version 2] doi: 10.1101/2023.05.10.23289777 (PMC11030495; doi:10.1101/2023.05.10.23289777)
Supplement: Supplement 2 [file NIHPP2023.05.10.23289777v2-supplement-2.pdf]

medRxiv preprint doi: https://doi.org/10.1101/2023.05.10.23289777; this version posted April 10, 2024. The copyright holder for this preprint (which was not certified by peer review) is the author/funder, who has granted medRxiv a license to display the preprint in perpetuity.
It is made available under a CC-BY 4.0 International license .

Supplemental Figures:

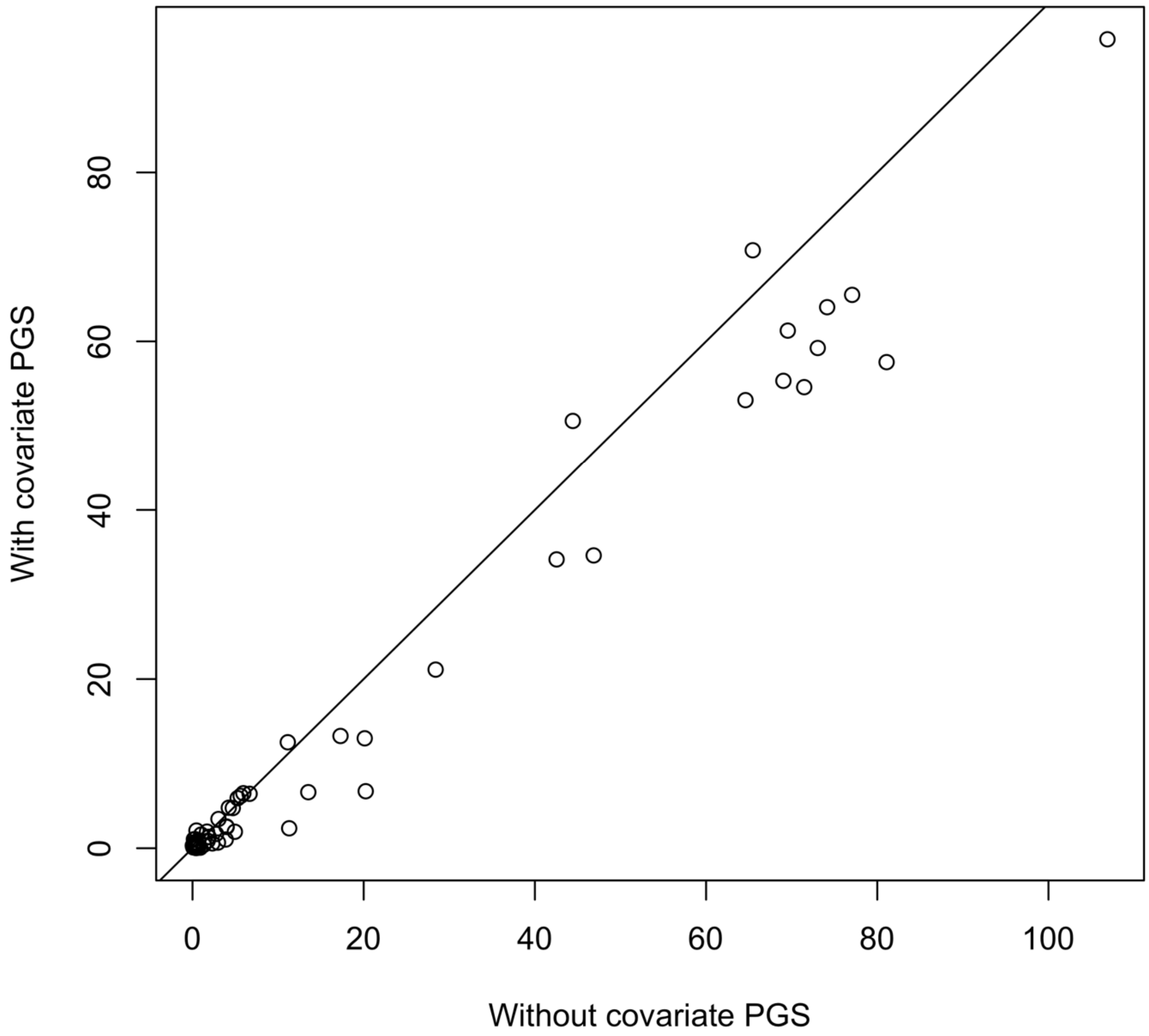

S Figure 1. PGS-covariate interaction term $-\log_{10}$(p-values) in UKBB EUR, with and without including the covariate PGS in the model – the mean $-\log_{10}(p)$ is reduced from 18.0899 to 14.97072 with their inclusions. Note age and sex PGS were not calculated, and their interaction p-values are excluded from this figure.

medRxiv preprint doi: https://doi.org/10.1101/2023.05.10.23289777; this version posted April 10, 2024. The copyright holder for this preprint
(which was not certified by peer review) is the author/funder, who has granted medRxiv a license to display the preprint in perpetuity.
It is made available under a CC-BY 4.0 International license .

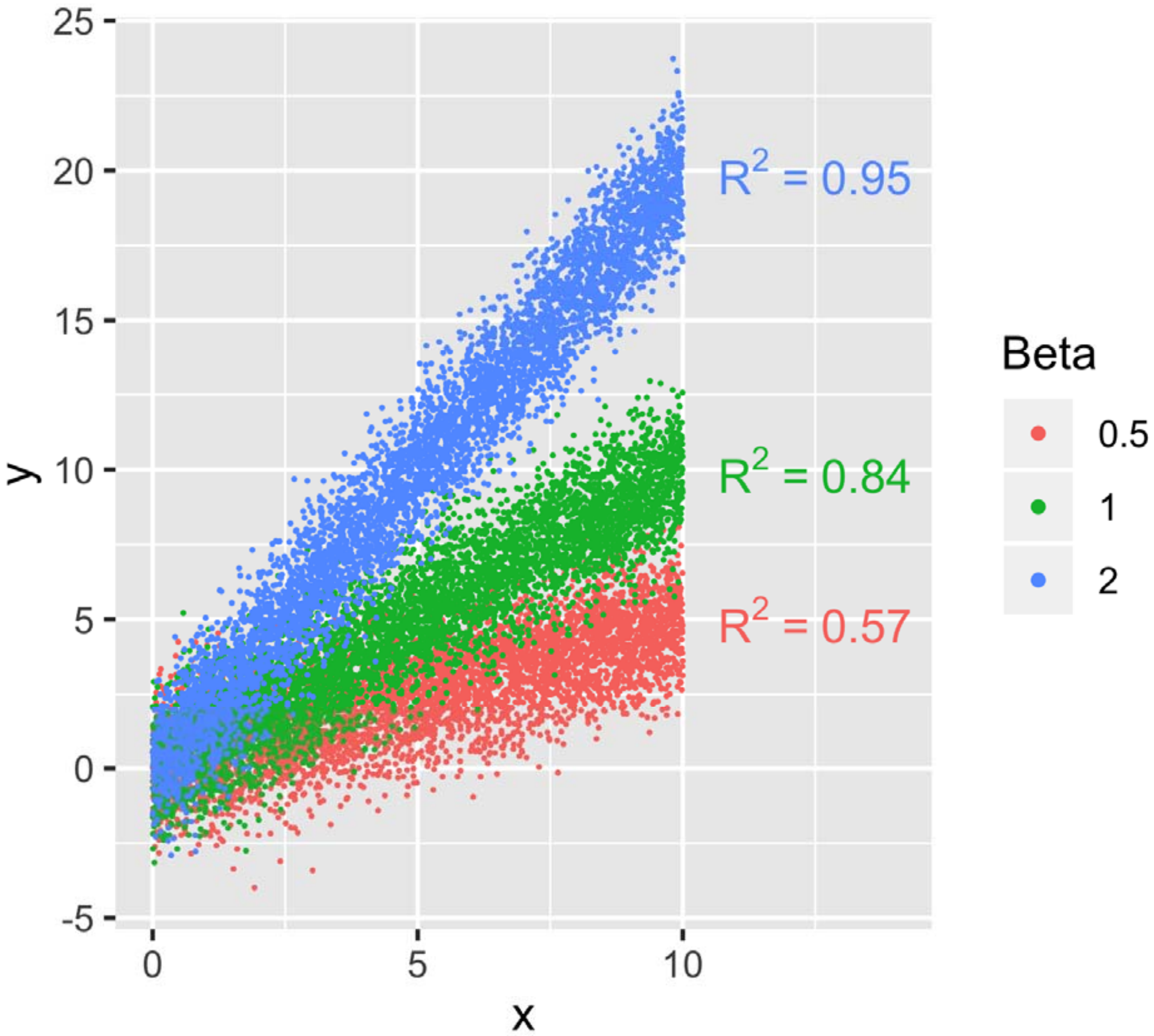

S Figure 2. Three sets of simulated data with varying regression line slopes, showing how model R^2 changes when regression line slope changes, all else being equal. Residuals were sampled from a normal distribution (mean=0, sigma=sqrt(π/2)) to give mean squared error=1. 5,000 x-values were sampled for each line, uniformly distributed from 0-10. Despite having the same mean squared error, model R^2 increases as beta increases.

medRxiv preprint doi: https://doi.org/10.1101/2023.05.10.23289777; this version posted April 10, 2024. The copyright holder for this preprint (which was not certified by peer review) is the author/funder, who has granted medRxiv a license to display the preprint in perpetuity.
It is made available under a CC-BY 4.0 International license .

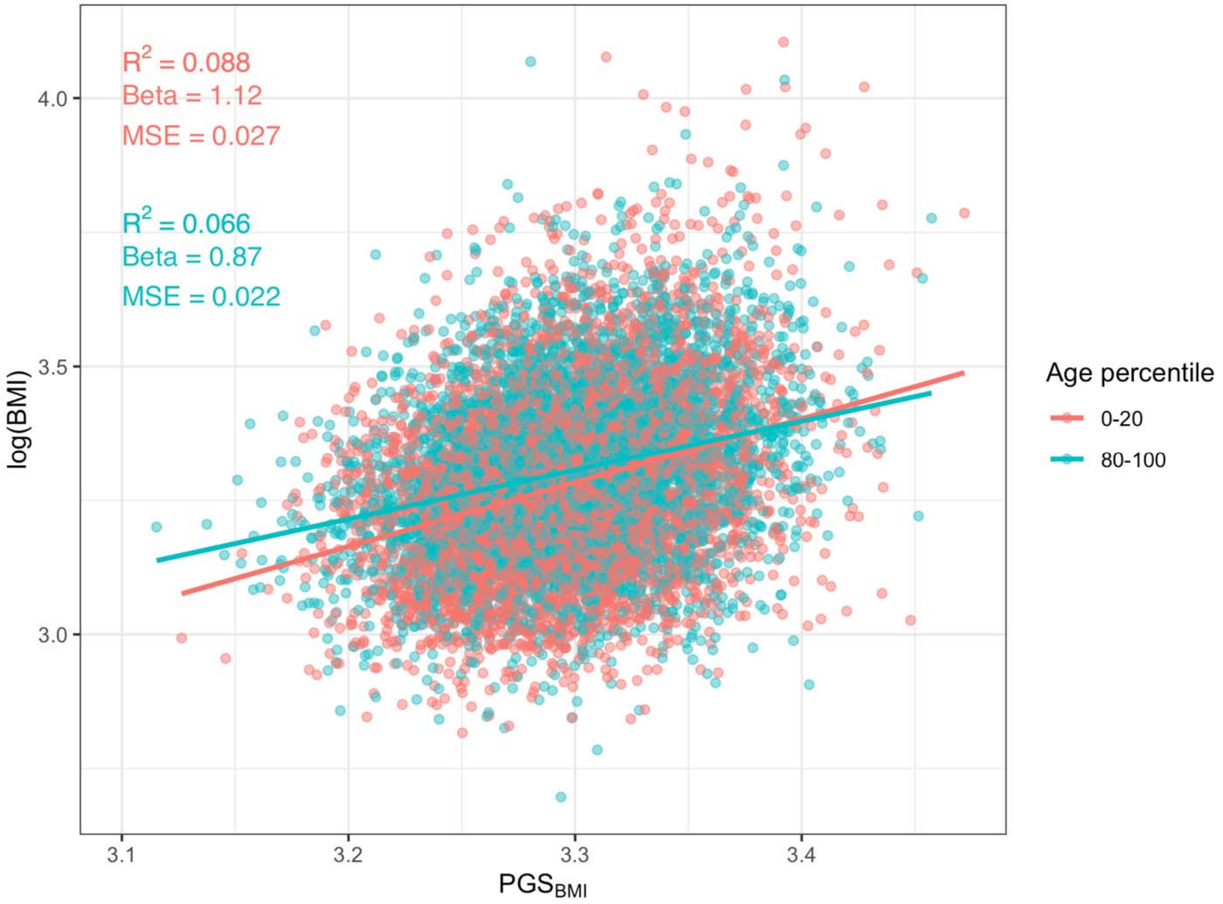

S Figure 3. Univariable association of PGS_{BMI} and log(BMI) in European UKBB, separately for the bottom and top quintiles of age. R^2 is higher in younger individuals, which is partially a consequence of the larger effect (as shown in S Figure 2), despite the mean squared error actually being higher.
